# Traumatic Tympanic Bulla Fracture in a Cat With Severe Head Trauma

**DOI:** 10.3389/fvets.2020.00372

**Published:** 2020-08-07

**Authors:** Fidel Sanroman-Llorens, Ana Whyte, Pedro Godinho, Enrique Castells, Eduardo Fraga

**Affiliations:** ^1^Centro Clínico Veterinario de Zaragoza, Zaragoza, Spain; ^2^Small Animal Hospital, Zaragoza Veterinariy College, Zaragoza, Spain; ^3^Hospital Veterinario Puchol, Madrid, Spain; ^4^Hospital Veterinario Bluecare, Málaga, Spain

**Keywords:** CT, bulla, fracture, cat, case report

## Abstract

A nine-year-old male European shorthair cat was referred to our practice with severe head trauma after suffering a road traffic accident (RTA). The patient presented marked facial swelling and multiple skin wounds and bruising, inspiratory dyspnea, palpable mandibular and maxillary fractures, serosanguinolent oronasal discharge and right eye exophthalmos and buphthalmos with loss of menace and pupillary reflex. After stabilizing the patient, a CT scan was performed under general anesthesia and an oesophagostomy tube was placed. The scan revealed the presence of multiple right tympanic bulla fractures. Multiple mandibular, maxillary, and palatine fractures were also present. The cat underwent surgery. Mandibular symphyseal separation and maxillary fractures were stabilized using intraoral cerclage wire fixation reinforced with composite and the right eye was enucleated. The rest of the fractures were treated conservatively. A CT scan 4 months after the trauma was also performed. At this point, the maxillofacial fractures were healing properly, and a bone callus demonstrating fusion of fragments of the right tympanic bulla was evident. There was absence of abnormal content inside the right tympanic bulla. The patient recovered uneventfully with no neurological deficits. To the author's knowledge this is the first case reporting a traumatic tympanic bulla fracture in the cat with case follow up, and the first case reported using CT as diagnostic imaging test.

## Introduction

Tympanic bulla injuries in small animals are commonly associated with neoplastic or infectious causes, or secondary to known surgical intervention such as ventral bulla osteotomy, or lateral bulla osteotomy (1–3). However, traumatic tympanic bulla fractures seem to be very uncommon in veterinary medicine. Rubin et al. reported one case presenting traumatic tympanic bulla fracture in a dog (4) and Krotscheck et al. one case in a cat (5), which lacks the case follow up and only use plain x-rays as diagnostic imaging test.

The presence of traumatic tympanic bullae fractures in whale skulls has also been reported (6). Humans lack tympanic bulla, but fractures affecting the tympanic region of the temporal bone are much more common and well-described in the literature. They are frequently caused by severe blunt trauma and usually present with concurrent regional injury to vital structures (7). Most commonly physical findings associated with temporal bone fractures, occurring alone or in combination, that have been previously described include hemotympanum, bleeding from the ear canal, tympanic membrane perforation, facial paralysis, and cerebrospinal fluid otorrhea (8). Critical intracranial injuries can be also found in these patients, including subdural hemorrhage, brain contusion and cerebral oedema (7). Imaging techniques play an important role in the evaluation of temporal bone trauma. Temporal bone anatomy is complex, consisting of numerous small, important and anatomically close structures. Certain imaging findings can significantly change patient management or change surgical approach (9). Long-term close follow-up is also mandatory because the late occurrence of potentially life-threatening complications, including meningitis (10). It is unknown whether the risk of concurrent intracranial injury and management strategies for tympanic bulla fracture in small animals can be appropriately derived from the approach to equivalent injuries in humans (4).

## Case Description

**Figure 1:**
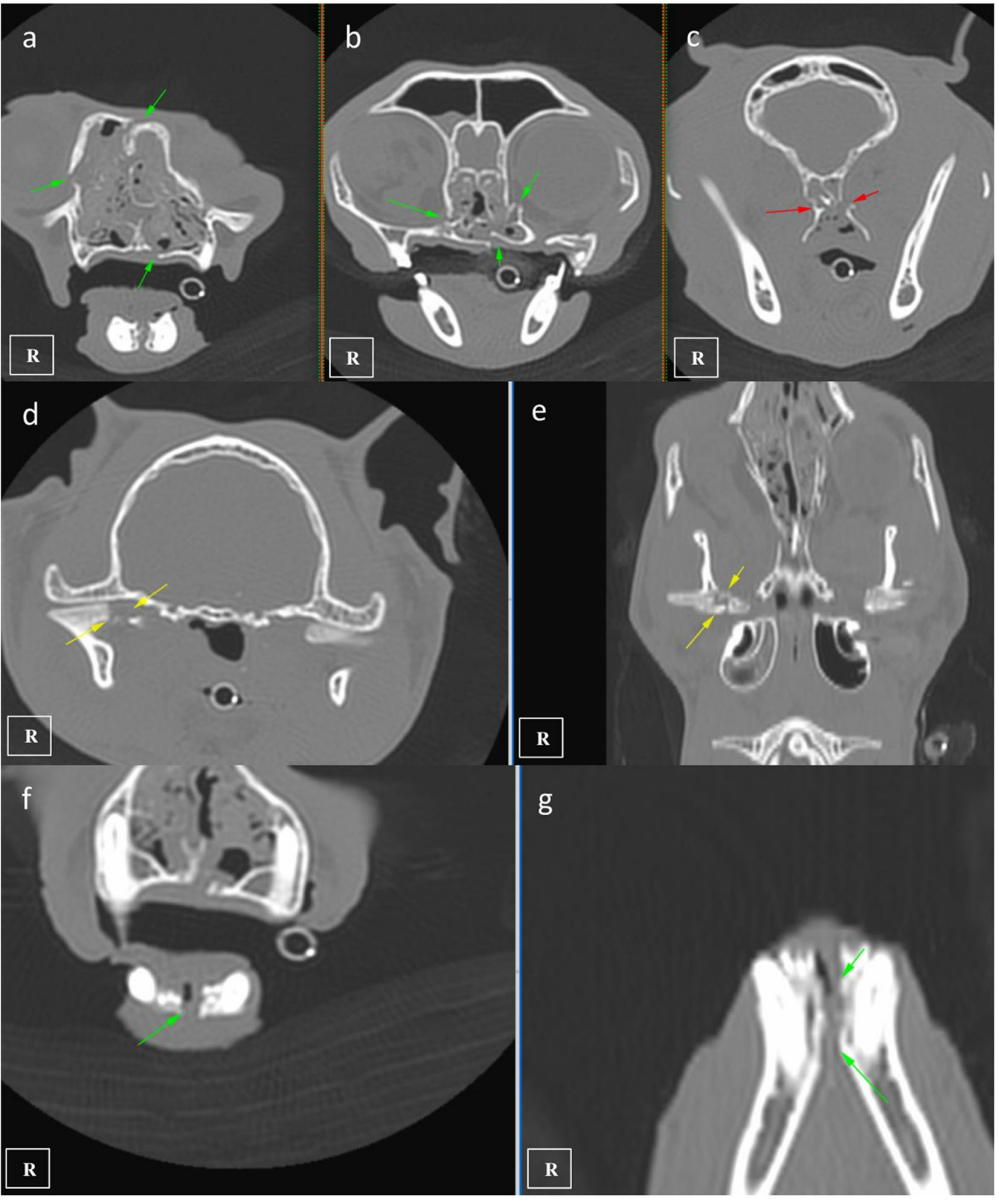
CT images showing multiple maxillofacial fractures affecting the maxillary and palatine bones (**a,b** green arrows), pterygoid hamulus (**c** red arrows), mandibular right condyle (**d,e** yellow arrows), and mandibular symphyseal separation (**f,g** green arrows).

**Figure 2:**
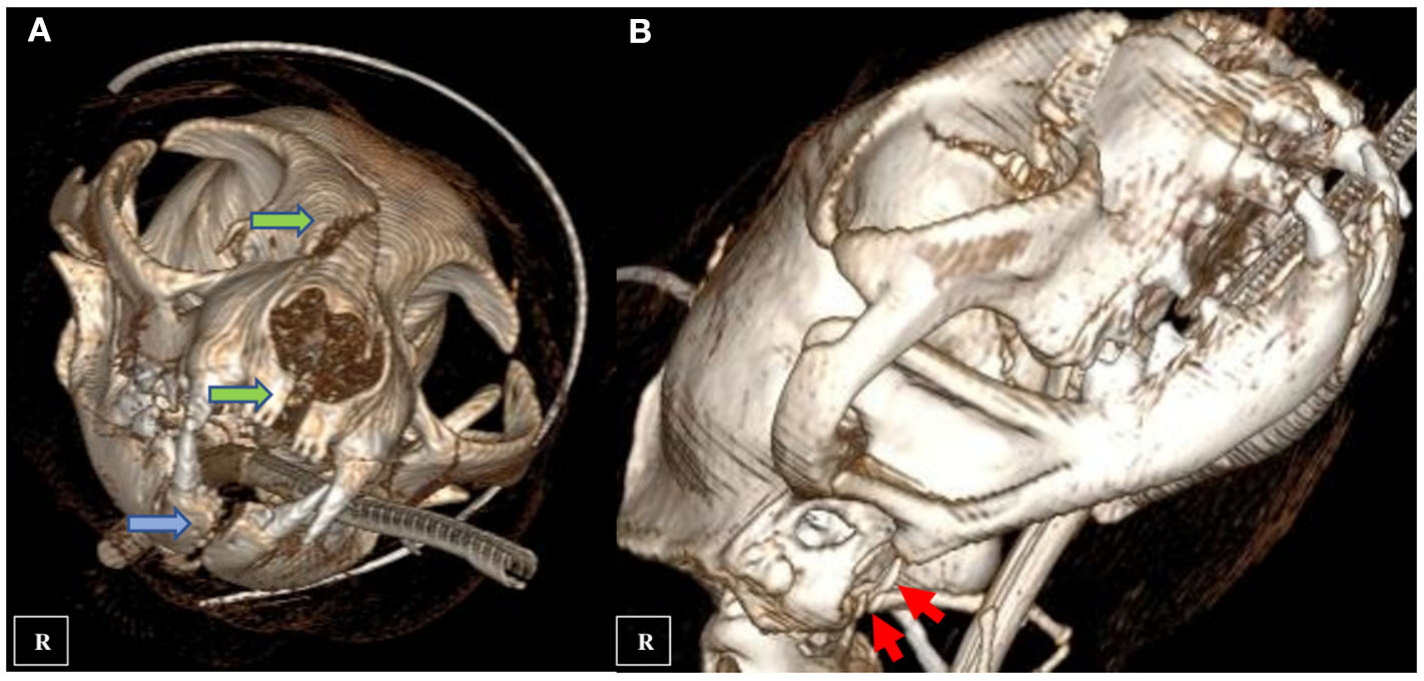
CT three-dimensional reconstruction showing multiple maxillary fractures (**A** green arrows), mandibular symphyseal separation (**A** blue arrow) and comminute fracture of the right tympanic bulla (**B** red arrows).

**Figure 3:**
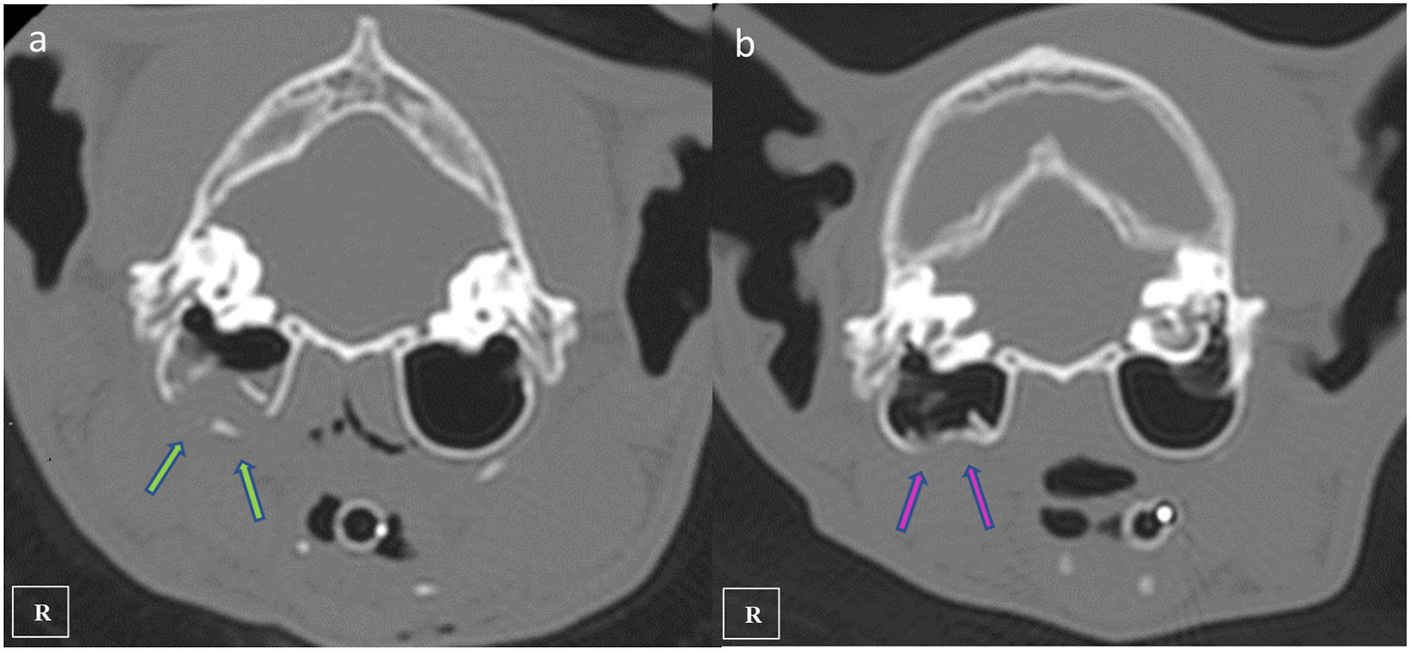
Transverse plane CT images showing the right comminuted tympanic bulla fracture at initial presentation (**a** green arrows) and fracture healing observed 4 months after the diagnostic CT scan (**b** pink arrows).

A nine-year-old male European shorthair cat was admitted presenting severe head trauma after suffering a RTA. Upon presentation, the patient showed marked inspiratory dyspnea, facial swelling and multiple skin wounds and bruising, right eye exophthalmos and buphtalmos, serosanguinolent oronasal discharge, multiple palpable mandibular, maxillary fractures and a maxillary right canine tooth crown fracture. Oxygen was administered and an IV catheter placed. Intravenous crystalloid fluid therapy (2 mL/kg/h 0.9% NaCl), 0.2 mg/kg methadone (Semfortan; Eurovet Animal Health BV), and 22 mg/kg cephazoline (Cefazolina; Laboratorios Normon) were also administered intravenously. Thoracic radiographs showed a mixed interstitial and alveolar pattern compatible with a mild pulmonary contusion affecting both cranial lobes. Serum biochemistry and complete blood count were performed and showed mild anemia and neutrophilia and a moderate increase of the ALT (alanine aminotransferase) value. Initial neurological examination was then performed and showed present but reduced movement of facial muscles, right ear and right nostril, as well as a slight facial asymmetry with right ear and lip drooping: these findings were consistent with a mild paralysis of the right facial nerve (facial paresis). Absent menace response and pupillary reflex of right eye was also observed. Complete right eye ophthalmologic examination also revealed exophthalmos, buphthalmos (40 mm Hg) and subconjunctival hemorrhage. The cat was hospitalized and treated with IV administration of fluid therapy (Ringer Lactate 2 ml/kg/h), 0.2 mg/kg methadone q6 h (Semfortan; Eurovet Animal Health BV) 0.05 mg/kg meloxicam q24 h (Metacam; Boehringer Ingelheim) and 22 mg/kg cephazoline q12 h (Cefazolina; Laboratorios Normon). One drop of dorzolamide 2% (Trusopt; Santen Pharmaceutical Spain S.L.) every 6 h and lubricant eye drops artificial tears every 2 h were applied to the right eye. Neuro-ophthalmic assessment was done every 6 h. Two days after admission a skull CT-scan (Brivo CT325; General Electrics Healthcare) was performed under general anesthesia. The CT images were acquired using bone and soft tissue algorithms, a voltage of 120 kV, an amperage of 80 mA and 1 mm slice thickness. Premedication consisted in 5 μg/kg dexmedetomidine (Dexdomitor; Esteve S.A) and 0.2 mg/kg methadone (Semfortan; Eurovet Animal Health BV) adm IV. Induction was performed using dose-effect propofol 10% (Lipuro; Bbraun) adm IV and anesthesia maintained using Isofluorane 2% (Isoflo; Esteve S.A.). An oesophagostomy tube was also placed under the same anesthetic procedure. Skull CT revealed bilateral maxillary and palatine fractures (Figures 1a,b), moderately displaced right eye exophthalmos and buphthalmos, bilateral fractures of pterygoid hamulus (Figure 1c), complete sagittal and comminuted fracture of the right mandibular condyle (Figures 1d,e), mandibular sympyseal separation (Figures 1f,g, 2A) and comminute fracture of the right tympanic bulla affecting its ventral aspect, which appeared partially collapsed with dorsal displacement of the fragments (Figures 2B, 3a). Fluid opacity content consistent with the serosanguinous nasal discharge was also observed in both nasal cavities, ventral aspect of both frontal sinuses and inside the right tympanic bulla. There were no signs of intracranial hemorrhage.

**Figure 4:**
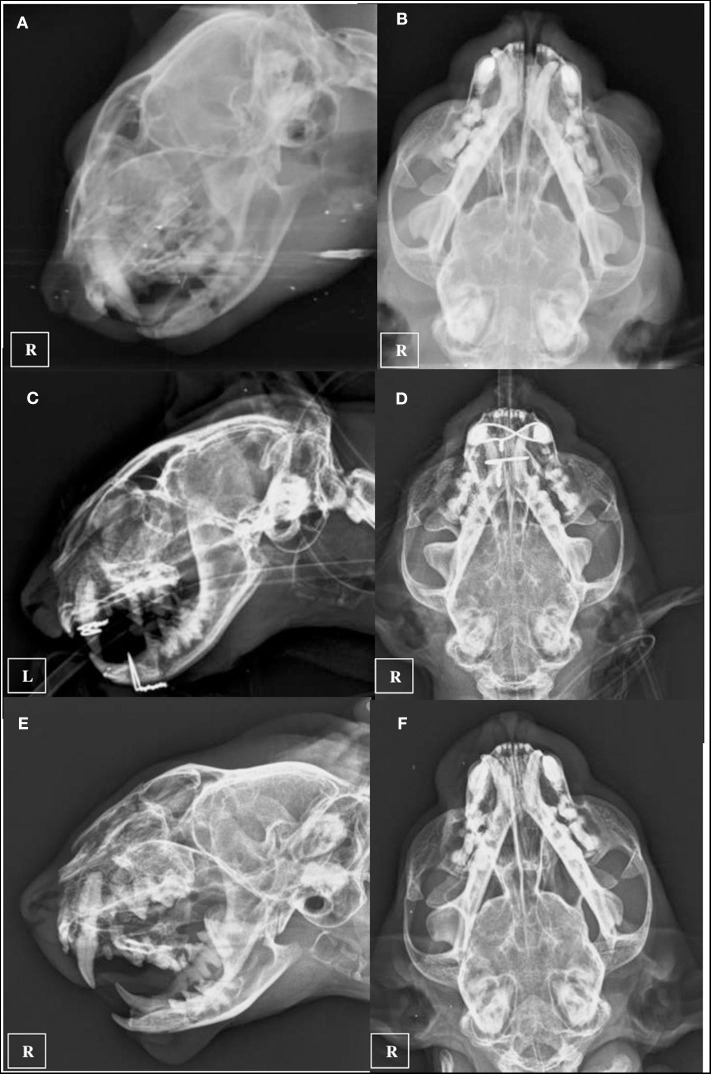
Ventro-dorsal and latero-lateral pre-operatory x-rays **(A,B)**, inmediate post-operatory x-rays **(C,D)**, and 5 weeks recheck x-rays after implant removal was performed **(E,F)**.

Two days following the initial general anesthetic event surgery was performed using the same anesthetic protocol. Maxillary fractures were stabilized using a single 1.0 mm cerclage wire placed in a figure-of-eight interdental wiring pattern which was reinforced using a composite splint. The mandibular symphyseal separation was fixed using a single loop of 1.0 mm cerclage wire (Figures 4C,D). The right eye was still buphthalmic before surgery, with absent menace response, pupillary reflex and an increased intraocular pressure of 36 mm Hg, so it was considered non-viable and enucleation was also performed under the same anesthetic procedure. The cat was hospitalized 2 days more and was discharged with an Elisabeth collar and the feeding tube kept in place. Postoperative medication consisted in 0,05 mg/kg Meloxicam (Metacam; Boehringer Ingelheim) and 20 mg/kg cephalexine (Rilexine; Virbac España S.A.) administered orally for 1 week, and soft diet was slowly encouraged and introduced. 10 days after surgery revealed that the inspiratory dyspnea had resolved, the cat was eating well and there was mild improvement of signs associated with facial nerve paralysis. The sutures and feeding tube were removed under light sedation using a combination of 5 mg/kg dexmedetomidine (Dexdomitor; Esteve S.A) and 0.1 mg/kg methadone (Semfortan; Eurovet Animal Health BV) administered intramuscularly. Five weeks later, neurologic examination showed no neurological deficits. The patient was sedated again with the same protocol and x-ray images were performed demonstrating continued bone healing with callus formation of maxillary and mandibular fractures were observed, so cerclage wires and composite were removed (Figures 4E,F). Four months after the trauma a control CT scan was performed using the same CT anesthetic and image acquisition protocol. At this recheck, the maxillofacial fractures were noted to have healed properly, and the bony callus was present demonstrating fusion of the right tympanic bulla fragments (Figure 3b). The previously noted fluid opacity in the right tympanic bulla appeared resolved. Final recheck was performed 6 months after the trauma. No neurological deficits were present and the cat had recovered uneventfully. Normal masticatory function and occlusion were achieved.

## Discussion

There have been only a very few reports describing traumatic tympanic bulla fractures in the veterinary literature (4–6). To the author's knowledge, there is only one case reported of traumatic tympanic bulla fracture in the cat (5), which lacks the case follow up and only use plain x-rays as diagnostic imaging test. That cat presented multiple fractures affecting the prominent ventral aspect of the tympanic bulla, which seems very similar morphologically to the tympanic bulla fractures presented in our case report. These injuries could be rare because the middle ear structures are heavily protected by the cranium dorsally and the mandible craniolaterally (4, 11), and might be produced produced as the result of traumatic events involving high-energy injuries that usually are associated with multiple maxillofacial fractures. Damage to the structures associated with the tympanic bulla can result in facial nerve paralysis and Horner's syndrome (4), because of the proximity of CN VII and the ocular sympathetic pathway (12–14). The cat reported by Krotscheck et al. presented with Horner's syndrome. Horner's syndrome may have been caused by direct trauma to the vagosympathetic trunk or indirect trauma from the fractured tympanic bulla to the vagosympathetic trunk, cranial cervical ganglion, or postganglionic fibers. Cervical trauma was not observed in the cat involved in this case report, suggesting that Horner's syndrome was most likely caused by trauma to the area of the tympanic bulla, cranial cervical ganglion, or postganglionic fibers (5). The presence of severe buphthalmos and exophthalmos in our clinical case could have masked Horner's Syndrome clinical signs, so it is impossible to determine whether the patient could have been affected. Rubin et al. reported in 2013 a traumatic tympanic bulla fracture in a Pekinese. That dog presented also multiple maxillofacial fractures and facial nerve paralysis. Skull CT revealed a mildly comminuted fracture at the junction between the tympanic, and squamous and petrous parts of the right temporal bone, with ventral displacement of the majority of the tympanic bulla from the remainder of the temporal bone. The horizontal ear canal was avulsed from the bulla and no longer in alignment with the external acoustic meatus. Given the extensive trauma to the region of the middle ear observed with diagnostic imaging, it was not surprising that Rubin et al.'s case developed permanent ipsilateral facial nerve deficits (4). Initial neurological examination in our patient showed only signs of mild facial nerve paralysis (facial paresis), with present but reduced movement of facial muscles, right ear and right nostril and slight facial asymmetry with right ear and lip drooping. Differential diagnosis of facial nerve paresis in small animals include myasthenia gravis, hypothyroidism, neoplasia of middle or inner ear, idiopathic facial paralysis, otitis media/interna, surgical or external trauma, brainstem disease and other generalized lower motor neuron diseases (15). Neurological signs resolved progressively over the next 6 weeks. Considering the patient history, diagnostic imaging test results and follow-up examinations, a traumatic etiology producing facial nerve neuritis/neuropraxia seems to be the more likely cause of these deficits. Brainstem auditory evoked response testing was not carried out, so hearing function and possible affection of the right VIII cranial nerve remains unknown in our patient.

CT is more sensitive than plain radiography for the identification of skull fractures in dogs and cats due to the complex anatomy of the skull and the lack of superimposition of structures and superior resolution achieved with CT (16, 17). Rotscheck et al. used plain x-rays to diagnose the tympanic bulla fracture employing open-mouth rostrocaudal, ventrodorsal, left ventral-right dorsal oblique, and right ventral-left dorsal oblique radiographic views (5). In our case, the referring veterinarian provided only two orthogonal radiograph views (Figures 4A,B). Right tympanic bulla fractures were not noted radiographically before performing the skull CT. This lead us to believe that tympanic bulla fractures might be more frequent and sometimes undiagnosed using conventional radiology. Nevertheless, Rebekah et al. performed a CT analysis of skull fractures in 75 cats and did not describe any tympanic bulla fractures (18). CT also provides additional information regarding fracture configuration and may be used to direct treatment planning (16, 19). Furthermore, in humans the importance of temporal bone fractures relates not only to functional deficits from injury to structures within the temporal bone but also to regional and intracranial injuries, including subdural hemorrhage, brain contusion and cerebral oedema (7). CT did not show signs of intracranial injuries in our patient. The use of complementary MRI has been also advocated in fractures of the tympanic part of the temporal bone in human patients with facial nerve injury (20), and it might be also useful in veterinary medicine.

Due to the mild nature of the neurologic deficits and the risk of possible surgical complications, tympanic bulla fracture in this case was treated conservatively. However, surgical exploration, facial nerve decompression if needed and bulla fragment removal might have been the only realistic option in the authors' opinion. Maxillary and mandibular fractures were numerous but relatively stable, and were treated using intraoral fixation consisting in cerclage wires and acrylic composites. We favored this approach to provide a much less aggressive treatment and a shorter anesthetic episode in a patient with severe head trauma. Use of osteosynthesis plates to treat similar maxillary fractures has also been widely used (21–23). Right mandibular condylar fracture was successfully treated conservatively, with no crepitus and normal range of motion of the temporomandibular joint at the final recheck. Although the cat was able to eat normally and free of pain during exploration 6 months after the trauma, the owner was warned that mandibular condylectomy could be necessary in the future. The maxillary right canine tooth crown fracture was not treated initially because we decided to focus on the other maxillary fractures and because the fractured tooth was used as support for the figure-of-eight cerclage wire reinforced with composite. Canine tooth endodontics was recommended after implant removal but the owner declined to perform the procedure at that moment as he preferred to consider it lately depending on associated clinical signs if present. Right eye enucleation was performed as it was considered non-viable.

## Conclusion

Traumatic tympanic bullae fractures seem to be rare in small animals and associated with neurological deficits and severe head trauma. Although conservative management of the tympanic bulla fractures was successful in this patient, a larger review of the findings of these cases are required to establish optimal treatment guidelines.

## Data Availability Statement

The original contributions presented in the study are included in the article/Supplementary Material, further inquiries can be directed to the corresponding author/s.

## Ethics Statement

Written informed consent was obtained from the owners of the animals for the publication of this case report.

## Author Contributions

FS-L and AW: surgery and article writing. PG: article writing and clinical support. EC: CT scan procedure, anesthesia, and article writing. EF: CT scan report and article writing. All authors contributed to the article and approved the submitted version.

## Conflict of Interest

The authors declare that the research was conducted in the absence of any commercial or financial relationships that could be construed as a potential conflict of interest.
