# Supplementary figures and images for: Traumatic Tympanic Bulla Fracture in a Cat With Severe Head Trauma
Source: Front Vet Sci. 2020 Aug 7;7:372. doi: 10.3389/fvets.2020.00372 (PMC7426505; doi:10.3389/fvets.2020.00372)

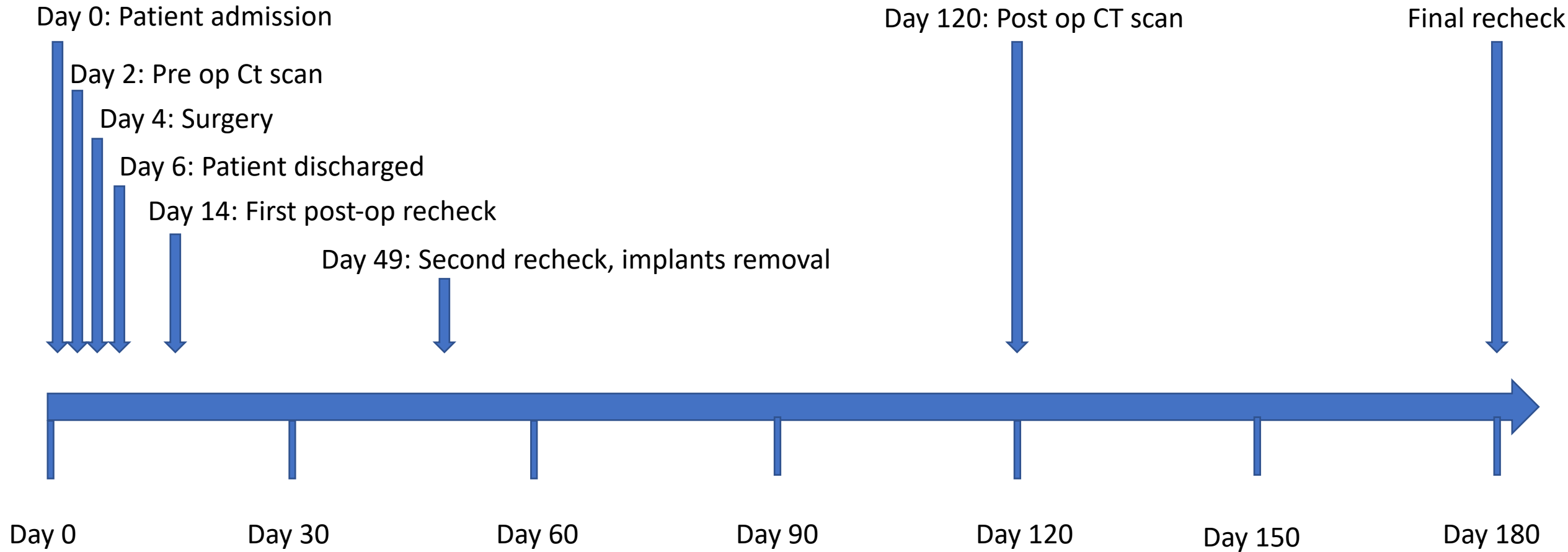

Supplement: Supplementary file 1 [file Data_Sheet_1.PDF]

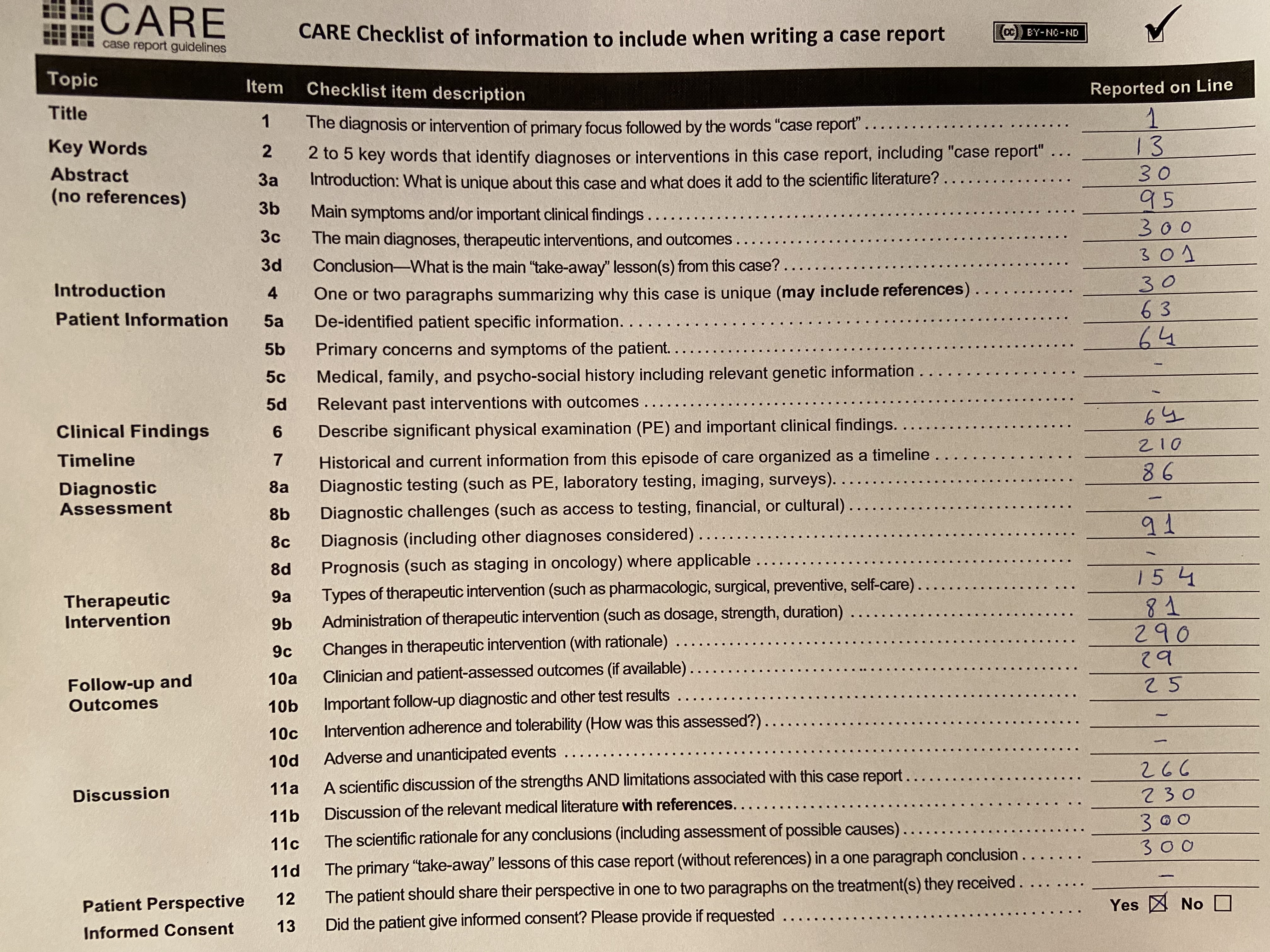

Supplement: Supplementary file 2 [file Image_1.JPEG]

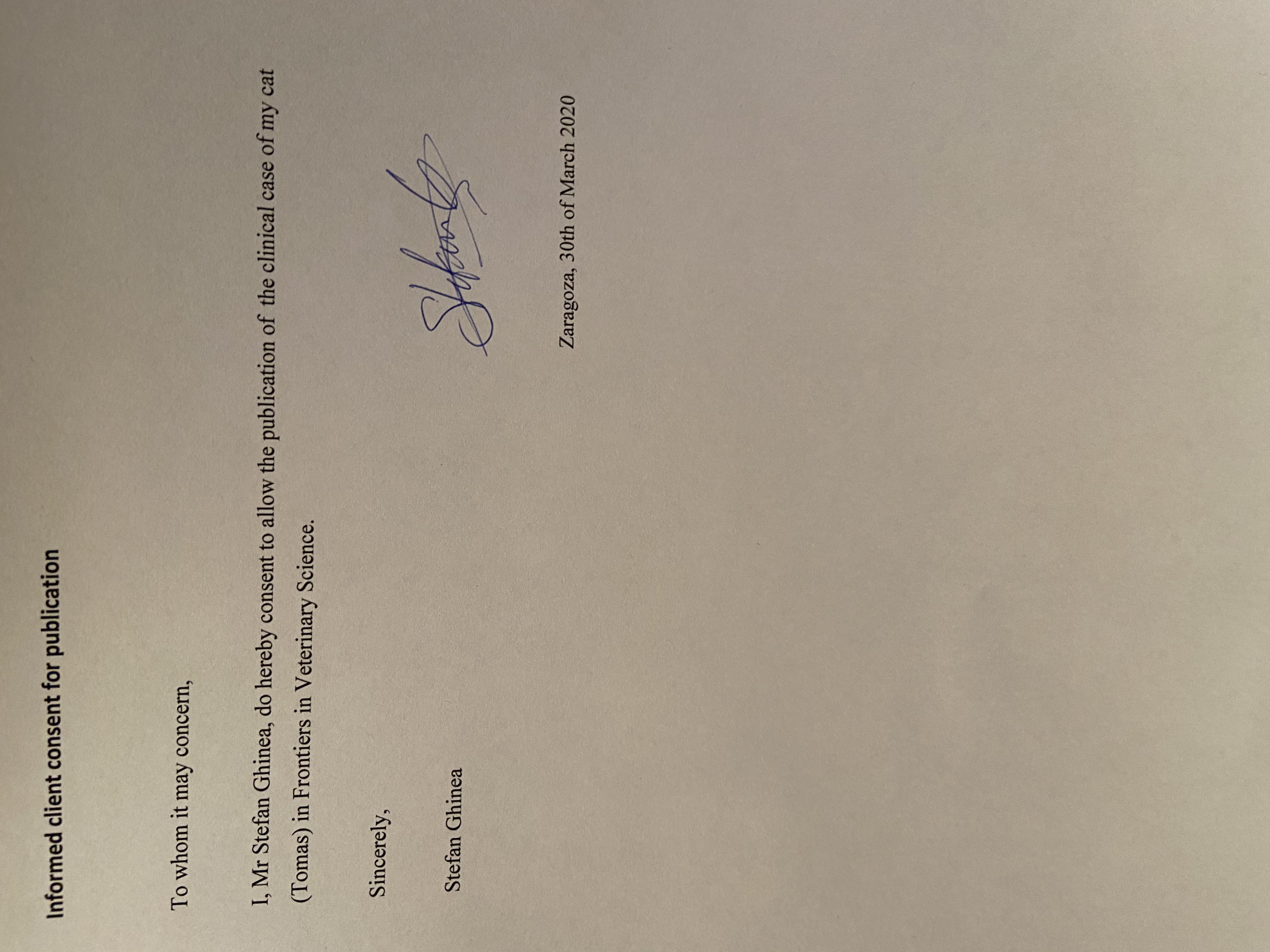

Supplement: Supplementary file 3 [file Image_2.JPEG]
